# Rationale and study design of PROVHILO - a worldwide multicenter randomized controlled trial on protective ventilation during general anesthesia for open abdominal surgery

**DOI:** 10.1186/1745-6215-12-111

**Published:** 2011-05-06

**Authors:** Sabrine NT Hemmes, Paolo Severgnini, Samir Jaber, Jaume Canet, Hermann Wrigge, Michael Hiesmayr, Edda M Tschernko, Markus W Hollmann, Jan M Binnekade, Göran Hedenstierna, Christian Putensen, Marcelo Gama de Abreu, Paolo Pelosi, Marcus J Schultz

**Affiliations:** 1 Department of Intensive Care Medicine & Laboratory of Experimental Intensive Care and Anesthesiology, Academic Medical Center, University of Amsterdam, The Netherlands; 2 Department of Environment, Health and Safety, University of Insubria, Varese, Italy; 3 Department of Critical Care Medicine and Anesthesiology (SAR B), Saint Eloi University Hospital, Montpellier, France; 4 Department of Anesthesiology, Hospital Universitar I Germans Trias I Pujol, Barcelona, Spain; 5 Department of Anesthesiology and Intensive Care Medicine, University of Leipzig, Germany; 6 Division Cardiac-, Thoracic-, Vascular Anesthesia and Intensive Care, Medical University, Vienna, Austria; 7 Department of Medical Sciences, Section of Clinical Physiology, University Hospital, Uppsala, Sweden; 8 Department of Anesthesiology and Intensive Care Medicine, University Hospital Carl Gustav Carus, Dresden, Germany; 9 Department of Anesthesiology, University of Bonn, Bonn, Germany; 10 Department of Surgical Sciences and Integrated Diagnostics, University of Genoa, Genoa, Italy

## Abstract

**Background:**

Post-operative pulmonary complications add to the morbidity and mortality of surgical patients, in particular after general anesthesia >2 hours for abdominal surgery. Whether a protective mechanical ventilation strategy with higher levels of positive end-expiratory pressure (PEEP) and repeated recruitment maneuvers; the "open lung strategy", protects against post-operative pulmonary complications is uncertain. The present study aims at comparing a protective mechanical ventilation strategy with a conventional mechanical ventilation strategy during general anesthesia for abdominal non-laparoscopic surgery.

**Methods:**

The PROtective Ventilation using HIgh versus LOw positive end-expiratory pressure ("PROVHILO") trial is a worldwide investigator-initiated multicenter randomized controlled two-arm study. Nine hundred patients scheduled for non-laparoscopic abdominal surgery at high or intermediate risk for post-operative pulmonary complications are randomized to mechanical ventilation with the level of PEEP at 12 cmH_2_O with recruitment maneuvers (the lung-protective strategy) or mechanical ventilation with the level of PEEP at maximum 2 cmH_2_O without recruitment maneuvers (the conventional strategy). The primary endpoint is any post-operative pulmonary complication.

**Discussion:**

The PROVHILO trial is the first randomized controlled trial powered to investigate whether an open lung mechanical ventilation strategy in short-term mechanical ventilation prevents against postoperative pulmonary complications.

**Trial registration:**

ISRCTN: ISRCTN70332574

## Background

Mechanical ventilation is a life-saving strategy in patients with respiratory failure. There is unequivocal evidence that mechanical ventilation in critically ill patients has the potential to aggravate or even initiate lung injury [1,2]. Patients with acute lung injury (ALI) could benefit from measures that prevent repeated collapse and re-expansion of alveoli, including the so-called open lung mechanical ventilation strategy with the use of higher levels of positive end-expiratory pressure (PEEP) and recruitment maneuvers [3]. Meta-analysis suggest this approach can waive the need for rescue therapies due to life-threatening hypoxemia [1], and even reduce mortality in patients with more severe ALI [4].

Mechanical ventilation is frequently mandatory in patients who undergo surgery. The effects of short-term intra-operative mechanical ventilation on pulmonary integrity are less well defined [5]. In addition, it is uncertain whether ventilation strategies that use higher levels of PEEP and recruitment maneuvers during the intra-operative period are beneficial in these patients [6,7]. However, higher levels of PEEP could reduce intra-operative atelectasis, decreasing repetitive collapse and re-expansion of dependent lung parts, and thereby attenuating pulmonary inflammation and coagulation [8,9]. Use of recruitment maneuvers to open the lungs has been found to improve the effectiveness of PEEP with regard to gas exchange during general anesthesia [10]. Intra-operative use of PEEP does not represent a common practice. Indeed, an observational study conducted in 28 centers in France revealed that most patients undergoing general surgery were ventilated without PEEP [11]. Thus, the intra-operative use of PEEP cannot be seen as clinical standard.

Post-operative pulmonary complications, in particular after general anesthesia >2 hours for abdominal surgery, add to the morbidity and mortality of surgical patients [12,13]. We hypothesize that a lung-protective mechanical ventilation strategy with higher levels of PEEP and recruitment maneuvers attenuates post-operative pulmonary complications in patients without lung injury (i.e., patients who do not fulfill the criteria for ALI at the moment of surgery).

PROVHILO aims at comparing the effects of such protective strategy and conventional mechanical ventilation in biomarkers of lung injury, post-operative pulmonary complications, extra-pulmonary complications and length of hospital stay in patients undergoing general anesthesia for open abdominal surgery.

## Methods

### Objectives and design

The PROtective Ventilation using HIgh versus LOw positive end-expiratory pressure ("PROVHILO") trial is a worldwide investigator-initiated multicenter randomized controlled two-arm trial.

The Institutional Review Board of the Academic Medical Center - University of Amsterdam, Amsterdam, The Netherlands, approved the trial. The PROVHILO trial is conducted in accordance with the declaration of Helsinki and was registered on October 29 2010 at http://www.controlled-trials.com with trial identification number ISRCTN70332574.

### CONSORT diagram

**Figure 1:**
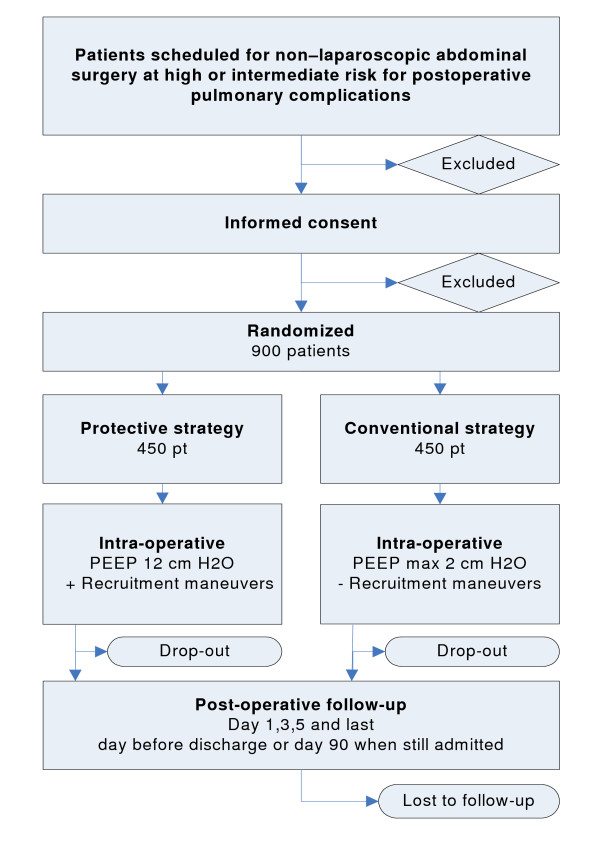
**CONSORT diagram of PROVHILO**. PEEP = positive end-expiratory pressure

Figure 1 shows the CONSORT diagram of the PROVHILO trial.

### Study population

Local investigators screen consecutive patients scheduled for non-laparoscopic abdominal surgery in participating centers worldwide. Demographic data on screened patients regardless of meeting enrolment criteria are recorded (registry: age, gender, type of surgery). A total of 900 patients are randomized to the 2 different mechanical ventilation strategies. In the participating centers at least 2 investigators are involved with the study. One researcher is involved with mechanical ventilation practice in the operation room, he/she will be blinded for the randomized intervention most closely to the time of tracheal intubation (depending on local situation) - the second investigator, blinded for randomization arm, will score the primary and secondary post-operative endpoints.

Patients with high or intermediate risk for post-operative pulmonary complications following non-laparoscopic abdominal surgery with general anesthesia are eligible for participation. To identify such patients the ARISCAT risk score (see Table 1) will be used [14]. This predictive risk index is developed by the ARISCAT Group to assess the individual pre-operative risk for post-operative pulmonary complications. An ARISCAT risk score ≥ 26 is associated with an intermediate to high risk for post-operative pulmonary complications.

**Table 1:** ARISCAT risk score - Independent predictors of risk for post-operative pulmonary complications identified in the logistic regression model

	Multivariate Analysis OR (95% CI) n = 1,624	β Coefficient	Risk Score†
Age (years)			
≤ 50	1		
51 - 80	1.4 (0.6 - 3.3)	0.331	3
>80	5.1 (1.9 - 13.3)	1.619	16
Pre-operative (SpO_2_, %)			
≥ 96	1		
91 - 95	2.2 (1.2 - 4.2)	0.802	8
≤ 90	10.7 (4.1 - 28.1)	2.375	24
Respiratory infection	5.5 (2.6 - 11.5)	1.698	17
in the last month			
Pre-operative anemia (≤ 10 g/dl)	3.0 (1.4 - 6.5)	1.105	11
Surgical incision			
Peripheral	1		
Upper abdominal	4.4 (2.3 - 8.5)	1.480	15
Intra-thoracic	11.4 (4.9 - 26.0)	2.431	24
Duration of surgery (hours)			
≤ 2	1		
2 - 3	4.9 (2.4 - 10.1)	1.593	16
>3	9.7 (4.7 - 19.9)	2.268	23
Emergency procedure	2.2 (1.0 - 4.5)	0.768	8

**High or intermediate risk for postoperative pulmonary complications following abdominal surgery: risk score ≥ 26**			

Abbreviations: CI, confidence interval; OR, odds ratio; SpO_2_, oxyhemoglobin saturation by pulse oximetry breathing air in supine position; g/dL, gram per decilitre † The simplified risk score was the sum of each logistic regression coefficient multiplied by 10, after rounding off its value

Patients planned for laparoscopic surgery are excluded from participation, as are non-adult patients (age <18 years), patients with a body mass index >40 kg/m^2^, pregnant patients (excluded by laboratory analysis), and patients who consented for another interventional study or decline to participate. In addition, patients who were on mechanical ventilation >30 minutes (e.g., because of general anesthesia for surgery) within last 30 days, are excluded. Other important exclusion criteria include: any previous lung surgery, history of previous severe chronic obstructive pulmonary disease (COPD) with (non-invasive) ventilation and/or oxygen therapy at home and/or repeated systemic corticosteroid therapy for acute exacerbations of COPD, ALI or acute respiratory distress syndrome expected to require prolonged post-operative mechanical ventilation, persistent hemodynamic instability or intractable shock (considered hemodynamic unsuitable for the study by the patient's managing physician), severe cardiac disease (New York Heart Association class III or IV, or acute coronary syndrome, or persistent ventricular tachyarrhythmia's), and recent immunosuppressive medication (receiving chemotherapy or radiation therapy within last 2 months).

All patients are asked for signed informed consent, as required by the institutional review board in accordance with the Declaration of Helsinki.

### Randomization and intervention

Randomization is performed using a dedicated, password protected, SSL-encrypted website. Randomization sequence is generated using random blocks and is stratified per center. No blocking is applied to other trial factors.

Patients are randomly assigned to mechanical ventilation with levels of PEEP at 12 cmH_2_O with the use of recruitment maneuvers (the lung-protective strategy) or mechanical ventilation with levels of PEEP at maximum 2 cmH_2_O without recruitment maneuvers (the conventional strategy). The PEEP level in the protective strategy is chosen to be 12 cmH_2_O, to achieve maximal interventional effect without causing harm to participating patients and to make the intervention acceptable for the participating clinicians. The conventional strategy is chosen based on a national survey in France that showed >90% of responding anesthetists to use levels of PEEP of 0 - 4 cmH_2_O without recruitment maneuvers [11]. Since not all available anesthesia ventilators can apply levels of PEEP <2 cmH_2_O, the level of PEEP is set at a maximum of 2 cmH_2_O with the conventional strategy. However the lowest possible level of PEEP is always chosen.

### Mechanical ventilation

Patients are ventilated with a volume-controlled mechanical ventilation strategy. Although it is left to the discretion of the attending anesthesiologist to use different fractions of inspired oxygen, it is advised to use at least 0.4, with the lowest oxygen fraction to maintain oxygen saturation ≥ 92%. The inspiratory to expiratory time ratio (I:E) is set at 1:2, and the respiratory rate is adjusted to reach normocapnia (end-tidal carbon dioxide partial pressure between 35 and 45 mmHg). Tidal volumes of <8 mL/kg predicted body weight (PBW) are advised to be used [15]. PBW is calculated according to a predefined formula: 50 + 0.91 × (centimeters of height - 152.4) for males and 45.5 + 0.91 × (centimeters of height - 152.4) for females [16,17]. Tidal volumes throughout this protocol refer to the actual inspired tidal volume in the ventilator circuit.

### Recruitment maneuver

Recruitment maneuvers, as part of the lung-protective strategy, are performed directly after intubation, after any disconnection from the mechanical ventilator, and directly before tracheal extubation. Recruitment maneuvers should not be performed when patients are hemodynamic unstable, as judged by the attending physician.

Recruitment maneuvers are not easily applied with available anesthesia ventilators since not all machines have an inspiratory hold function or other adequate facilities. To obtain standardization among centers, recruitment maneuvers are performed as follows:

1. peak inspiratory pressure limit is set at 45 cmH_2_O

2. tidal volume is set at 8 ml/kg PBW and respiratory rate at 6-8 breaths/min (or lowest respiratory rate that anesthesia ventilator allows), while PEEP is set at 12 cmH_2_O

3. inspiratory to expiratory ratio (I:E) is set at 1:2

4. tidal volumes are increased in steps of 4 ml/kg PBW until a plateau pressure of 30-35 cmH_2_O

5. 3 breaths are administered with a plateau pressure of 30-35 cmH_2_O

6. peak inspiratory pressure limit, respiratory rate, I:E, and tidal volume are set back to settings preceding each recruitment maneuver, while maintaining PEEP at 12 cmH_2_O

### Protocol drop-out

Anesthesiologists are allowed to change the ventilation protocol at any time point upon the surgeon's request, or if there is any concern about patient's safety. The level of PEEP can be modified according to the anesthesiologist in charge if the systolic arterial pressure drops <90 mmHg for more than 3 minutes despite intravenous fluid infusion and/or start of vasopressors, if dosages of vasopressors are at the highest level tolerated, if new arrhythmias develop which are unresponsive to treatment suggested by the Advanced Cardiac Life Support Guidelines [18], if there is need of massive transfusion to maintain Ht >21% (Hb >7 mg/dl), or if there is a surgical complication determining life-threatening situations.

### Rescue therapy

In both study groups, in case of desaturation (SpO_2 _<90%), after excluding airway problems, severe hemodynamic impairment and ventilator malfunction, a rescue strategy is proposed, which improves oxygenation with respectively a decreasing level of PEEP with increasing FiO_2 _in the lung-protective strategy group, and increasing levels of PEEP and FiO_2 _in the conventional group (see Table 2).

**Table 2:** Rescue therapies with the protective and the conventional strategy

Protective			Conventional		
**Step**	**FiO**_**2**_	**PEEP**	**Step**	**FiO**_**2**_	**PEEP**

1	0.5	12	1	0.5	2
2	0.5	10	2	0.6	2
3	0.5	8	3	0.6	3
4	0.5	6	4	0.6	4
5	0.6	6	5	0.6	5
6	0.7	6	6	0.7	5
7	0.8	6	7	0.8	5
8	0.8	4 or lower	8	0.8	6
			9	RM	6

See text for details.

Abbreviations: PEEP = positive end-expiratory pressure; FiO_2 _= fractional inspired oxygen; RM, recruitment maneuver

### Standard procedures

The study protocol stresses that routine general anesthesia, post-operative pain management, physiotherapeutic procedures and fluid management must be used in the peri-operative as well as the post-operative period according to each centers specific expertise and routine clinical use, to minimize interference with the trial intervention. However, it is suggested to perform post-operative pain management in order to achieve a visual analogue scale (VAS) pain score <3, to use regional or neuroaxial analgesia if indicated, to use physiotherapy by early mobilization, deep breathing exercises with and without incentive spirometry, and stimulation of cough in the post-operative period, to avoid fluid overload (according to the discretion of the responsible physicians) and to use appropriate prophylactic antibiotics when indicated. Data on the applied procedures will be collected and analysed.

### Follow up

Baseline variables are collected pre-operative at the pre-anesthetic visit or before induction of general anesthesia. The following variables are collected; gender, age, height, weight, functional status (independent, partially dependent or totally dependent), physical status (according to the American Society of Anesthesiologists (ASA), cardiac status (heart failure, according to the New York Heart Association (NYHA), acute coronary syndrome, or persistent ventricular tachyarrhythmia's),

COPD and use of inhalation therapy and/or steroids, respiratory infection in the last month, smoking status, alcohol status in the past 2 weeks, history of active cancer, weight loss >10% in the last 6 months, history of diabetes mellitus, use of oral anti-diabetics, use of antibiotics in the last 3 months, use of statins, type of scheduled surgery (emergency or non-emergency and surgical procedure), transfusion of blood products in the preceding 6 hours, vital parameters (timpanic temperature, respiratory rate, SpO2 (%), blood pressure, heart rate), airway secretion score (the patient is required to cough and the presence of secretion will be subjectively evaluated; if yes: purulent or not), VAS-scores for dyspnea and pain, blood samples (glycemia, uremia, creatinine, AST, ALT, bilirubin, Hb, WBC count, platelet count, PT, PTT, and biomarkers [see below]) and a chest X-ray (assessed on mono- and bilateral infiltrate, pleural effusion, atelectasis, pneumothorax, cardiopulmonary edema).

During the intra-operative period variables are recorded hourly after induction of anesthesia during the recruitment maneuver. These variables include duration and type of both anesthesia and surgical procedures, all administered drugs during anesthesia (e.g. anesthetics, vasoactive drugs, anti-arrhythmic medication), ventilator settings, vital parameters, fluid- and transfusion requirements, need of rescue therapy for hypoxemia and intra-operative complications possibly related to recruitment maneuvers (e.g. de-saturation, hypotension during recruitment maneuver, need for vasoactive medication).

Patients are assessed at the first five post-operative days and at the last day before discharge from the hospital. On day 90 hospital free-days are recorded; if the patient is still admitted to the hospital on day 90, this day will be recorded as last day of follow-up. Clinical data and the presence of pulmonary and extra-pulmonary postoperative complications are scored; the day of development of any complication is indicated. A chest X-ray will be taken on the first post-operative day, blood samples for laboratory tests (glycemia, uremia, creatinine, AST, ALT, bilirubin, Hb, WBC count, platelet count, PT, PTT) will be taken on day 1, 3 and 5 and blood samples for biomarkers are collected directly after surgery and on day 5. As mentioned above, one local investigator, blinded for randomization group will score the primary and secondary post-operative endpoints.

### Study endpoints

Primary endpoint - is a composed endpoint of all post-operative pulmonary complications with each complication weighing equally; it is presented as a total percentage of post-operative pulmonary complications. The post-operative complications are defined as: (a) mild respiratory failure (PaO_2 _<60 mmHg or SpO_2 _<90% in room air *but responding *to supplemental oxygen, (b) severe respiratory failure (need for non-invasive or invasive mechanical ventilation *or *a PaO_2 _<60 mmHg *or *SpO_2 _<90% *despite *supplemental oxygen), (c) development of ALI/ARDS (according to consensus guidelines [19]), (d) suspected pulmonary infection (patient receives antibiotics and meets at least one of the following criteria: new or changed sputum, new or changed lung opacities on chest X-ray when clinically indicated, tympanic temperature >38.3°C, WBC count >12,000/μl in the absence of other infectious focus), (e) pulmonary infiltrate (chest X-ray demonstrating unilateral or bilateral infiltrates), (f) pleural effusion (chest X-ray demonstrating blunting of the costophrenic angle, loss of the sharp silhouette of the ipsilateral hemidiaphragm in upright position, evidence of displacement of adjacent anatomical structures or (in supine position) a hazy opacity in one hemi-thorax with preserved vascular shadows), (g) atelectasis (suggested by lung opacification with shift of the mediastinum, hilum, or hemidiaphragm towards the affected area, and compensatory overinflation in the adjacent non-atelectatic lung), (h) pneumothorax (air in the pleural space with no vascular bed surrounding the visceral pleura), (i) bronchospasm (newly detected expiratory wheezing treated with bronchodilators), (j) aspiration pneumonitis (respiratory failure after the inhalation of regurgitated gastric contents), (k) cardiopulmonary edema (clinical signs of congestion, including dyspnea, edema, rales and jugular venous distention, with the chest X-ray demonstrating increase in vascular markings and diffuse alveolar interstitial infiltrates).

Secondary clinical endpoints - include (a) intra-operative ventilation strategy related complications (e.g. de-saturation, hypotension during recruitment maneuver, need for vasoactive medication), (b) unexpected need for ICU admission or ICU readmission, (c) hospital-free days at follow-up day 90, (d) post-operative wound healing and (e) post-operative extra-pulmonary complications. Extra-pulmonary complications include SIRS, sepsis, severe sepsis, septic shock (all according to consensus criteria [20]), extra-pulmonary infection (wound infection or any other infection), coma (Glasgow Coma Score <8 in the absence of therapeutic coma or sedation), acute myocardial infarction (according to universal definition of myocardial infarction [21]), acute renal failure (according to the RIFLE classification system [22]), disseminated intravascular coagulation (according to ISTH diagnostic scoring system for DIC [23]), gastro-intestinal failure (defined as; gastro-intestinal bleeding or gastro-intestinal failure according to GIF-score [24]) and hepatic failure (defined as; serum bilirubin level >2 mg/dL with elevation of the transaminase and lactic dehydrogenase levels above twice normal values).

Other study parameters - Blood samples will be collected and analyzed for systemic markers of lung injury (including but not limited to soluble Receptor for Advanced Glycation Endproducts (sRAGE), Clara Cell protein-16 (CC-16), surfactant proteins A and D and levels of proinflammatory and procoagulant/antifibrinolytic mediators (including but not limited to interleukin (IL)-6, IL-8, tumor necrosis factor (TNF)-α, and thrombin-antithrombin (TAT), protein C, and plasminogen activator inhibitor (PAI)-1). The abovementioned biomarkers of lung injury, acute inflammation and coagulopathy have been shown to correlate with poor clinical outcome in patients with ALI/ARDS [25]. Notably, with short-term mechanical ventilation rises in systemic levels of lung injury biomarkers [26], acute inflammation [9] and procoagulant/antifibrinolytic mediators [8] have been described. Lung-protective mechanical ventilation strategies attenuated the rise in levels of some of the abovementioned mediators in patients with ALI/ARDS [27], as well as patients who underwent short-term mechanical ventilation because of surgery [8,9]. Most of these trials compared the effect of different tidal volumes.

The injury induced by mechanical ventilation originates in the lung, but may also affect distal organs by release of mediators from the lung into the systemic circulation [28,29]. Therefore systemic biomarkers of distant organ injury, in particular the kidney, are determined (including, but not limited to neutrophil gelatinase-associated lipocalin (NGAL) and cystatin C).

### Statistical considerations

Sample size calculation - the required sample size is calculated from an estimated effect size derived from data collected in the ARISCAT study [14] and previous studies on the incidence of postoperative pulmonary complications [12,13,30]. A two group χ^2 ^test with a 0.05 two-sided significance level will have 80% power to detect the difference (in post-operative pulmonary complications) between conventional mechanical ventilation (24%) and open lung mechanical ventilation (16.5%) (Odds ratio of 0.626) when the sample size in each group is 450.

Interim analysis - one main concern is not to withhold positive effects of the open lung mechanical ventilation strategy to the control group. Therefore, interim analyses are performed after 300 and 600 patients. The first interim analysis is performed when 300 patients have successfully been included and followed-up. If the intervention has a strong trend for improving post-operative pulmonary complications (as defined above) with a *p*-value < 0.0005 is found at 300 patients or <0.014 at 600 patients, termination of the study is considered. The third and final analysis is performed at 900 patients with a *p*-value of 0.045 for significance. When post-operative pulmonary complications occur significantly more frequent in the intervention group, terminating the study due to harm will be considered when *p *≤ 0.022 for each interim analysis.

### Statistical analysis

Normally distributed variables will be expressed by their mean and standard deviation; not normally distributed variables will be expressed by their medians and interquartile ranges; categorical variables will be expressed as n (%). In test groups of continuous normally distributed variables, Student's *t-*test will be used. Likewise if continuous data are not normally distributed the Mann-Whitney *U *test will be used. Categorical variables will be compared with the Chi-square test or Fisher's exact tests or when appropriate as relative risks. Where appropriate statistical uncertainty will be expressed by 95% confidence levels.

Primary outcome is the total occurrence of pulmonary complications within the first 5 post-operative days, presented as a percentage. The percentage will be analyzed as continuous data. If the data is normally distributed, Student's *t-*test will be used or when not normally distributed the Mann-Whitney *U *test will be used.

As this is a randomized controlled trial, we expect that randomization in this large study population will sufficiently balance the baseline characteristics. Baseline balance is tested and imbalance compensated in all pre-operative variables and on ARISCAT scores [14] (as mentioned above). However if imbalance occurs, the confounding factor will be corrected using a multiple logistic regression model. For this we will treat the proportion as a binary response (complications occur during day one to day five post-operative).

Time to event variables (primary and secondary outcomes) are analyzed using a proportional hazard model adjusted for possible imbalances of patients' baseline characteristics. Time course variables (e.g. repeated measures of vital parameters, blood values, VAS-scores, actual mobility) are analyzed by a linear mixed model. The linear mixed models procedure expands the GLM so that the data are permitted to exhibit correlated and non-constant variability. The model includes two factors: 1) study group (fixed factor, intervention or control group), each level of the study group factor can have a different linear effect on the value of the dependent variable; 2) time as a covariate, time is considered to be a random sample from a larger population of values, the effect is not limited to the chosen times.

## Study Organization

The Executive Committee is constituted of the study principal investigator and the principal investigators of the investigating centers that approved the final trial design and protocol issued to the clinical sites and to the Data and Safety Monitoring Board (DSMB).

The independent DSMB watches over the ethics of conducting the study in accordance with the Declaration of Helsinki, monitors patient safety and reviews safety issues as the study progresses. All serious adverse events, and all unexpected and related or possibly-related adverse events will be reported blinded to the appointed international SAE-manager, who assesses the events and reports this information to the DSMB within 24 hours of that event in the case of a serious adverse event or within one week in the case of an adverse event.

The Steering Committee is composed of the principal investigators of the principle participating centers who contribute to the design and revisions of the study protocol.

The National Coordinators are responsible for administrative management and communication with the local principle investigator and provide assistance to the participating clinical sites in trial management, record keeping and data management.

## Discussion

It has become clear that mechanical ventilation can attenuate lung damage and may even be the primary factor in lung injury [2,3]. ALI/ARDS is characterized by heterogeneous distribution of pulmonary aeration. During ventilation the aerated part of the lung receives the largest part of the tidal volume, potentially causing overdistention with excessive alveolar wall tension and stress. The non-aerated atelectatic lung regions are prone to repeated collapse and re-expansion of alveoli, causing shear stress and diffuse mechanical damage of the alveoli [2,28]. This could trigger local and systemic inflammation, which has been suggested to cause ventilator-associated lung injury [1,8,9].

Protective mechanical ventilation using lower tidal volumes could reduce ventilator-associated lung injury. Indeed, the use of lower tidal volumes has been found beneficial in patients who needed long-term mechanical ventilation for ALI/ARDS [1,15]. Two retrospective studies [31,32] and one randomized controlled trial [33] suggest lower tidal volumes to be beneficial in patients without acute lung injury in long-term ventilation as well. Other trials suggest that ventilation with lower tidal volumes is also beneficial in short-term ventilation for patients without preexisting lung injury [8,9,34]. In these trials different levels of PEEP were used, making comparison and interpretation of the additional effect of PEEP difficult.

During general anesthesia reductions in end-expiratory lung volume and increases in airway closure is commonly seen [35]. Both contribute to atelectasis formation. The most important morbid post-operative pulmonary complication is atelectasis formation, which increases the risk for pneumonia and hypoxic acute respiratory failure [36]. Post-operative pulmonary complications, in particular post-operative respiratory failure, add to the morbidity and mortality of surgical patients [12,13]. PEEP prevents alveolar collapse and atelectasis formation. Recruitment maneuvers can be used to achieve initial alveolar recruitment [3,37]. Data suggests that recruitment maneuvers adequately support the beneficial effects of PEEP in short-term ventilation [38,39]. However, PEEP levels should not be too high, to avoid overdistention of the lung [1,40].

Various studies showed mechanical ventilation according to an open lung concept to improve ventilatory efficacy of the lungs in patients with healthy lungs undergoing general anesthesia [3,37]. Studies have shown the open lung concept to attenuate inflammatory responses and to prevent loss of functional residual capacity in cardiac surgery patients [34,41]. Of note, there is some controversy about the clinical importance of the cyclic collapse of alveoli. Indeed, the potential of ventilation strategies with lower tidal volumes and PEEP for protecting the lungs during the intra-operative period in patients without previous lung injury has been questioned [6,7].

The PROVHILO trial is the first randomized controlled study powered to investigate whether protective mechanical ventilation using higher levels of PEEP complemented by recruitment maneuvers attenuates post-operative pulmonary complications. The two ventilation strategies used in the PROVHILO trial are composed to match as many clinically applied anesthesia ventilators as possible. With these standardized ventilation strategies, we aim to minimize variation between ventilation strategies used in the participating centers.

The primary endpoint of this trial is a composed endpoint (post-operative pulmonary complications). This could be seen as a shortcoming, since the effect of the intervention on one post-operative pulmonary complication could be diluted if other post-operative pulmonary complications are not affected, or affected to a lesser content. However, since we collect and report on all post-operative pulmonary complications, it may still be possible to determine the effects on separate complications.

The main concern in the statistical interim analysis is not to withhold positive effects of the treatment to the control group. However, to achieve maximal protection for patients and to have a lower chance of achieving positive effects of the intervention on post-operative pulmonary complications if they were not really present, different stopping rules are defined for a strong beneficial effect on post-operative pulmonary complications of the intervention versus a worse effect on post-operative pulmonary complications.

The spectrum of ventilator-associated lung injury does not only include pulmonary inflammation, but also an increase in systemic inflammatory mediators [2,42-44]. The lung has been suggested as an important causative part of the inflammation-induced systemic disease state that can evolve to multi-organ failure, rather than merely a pulmonary disease process. Alveolar collapse during mechanical ventilation can lead to activation of inflammatory response both locally and systemically, which can play a role in modulating the individual patient's outcome [3,28,45]. To determine this possible effect on patients in this trial, secondary endpoints on extra-pulmonary complications are collected and reported, as well as blood samples for the determination of specific markers of distal organ injury.

Several confounding factors can be suggested. Post-operative pain is a commonly acknowledged contributor to post-operative atelectasis [46,47]. Respiratory chest physiotherapy has been shown to decrease postoperative respiratory complications in cardiac surgery, when performed before surgery [48]. It is still uncertain if post-operative physiotherapeutic procedures are beneficial, although there is some evidence in favor of physiotherapy [47]. Excessive intra-operative fluid administration is another possible contributing factor to the development of respiratory failure [49]. These factors are not protocolized by the PROVHILO trial. The protocol stresses that general anesthesia, post-operative pain management, physiotherapeutic procedures, fluid management and all other peri-operative procedures are to be performed according to the centers' specific expertise and routine clinical use. We aim to minimize interference with the effect of PEEP and recruitment maneuvers on post-operative pulmonary complications. Suggestions on the abovementioned peri-operative procedures are made in the protocol, to keep the variability as small as possible. No suggestions are made on type of anesthesia to use, to make the trial as accessible as possible for anesthesiologists. It is known, however, that several anesthetic drugs affect lung capacity during surgery [50,51]. Since we collect and report on all commonly known risk factors for post-operative pulmonary complications and intra-operative administered drugs, it may still be possible to determine the effect on the primary and secondary outcomes.

In conclusion, the PROVHILO trial is a worldwide investigator-initiated randomized controlled trial powered to test the hypothesis that an open lung mechanical ventilation strategy using higher levels of PEEP and recruitment maneuvers during short-term intra-operative mechanical ventilation prevents against post-operative pulmonary complications. The PROVHILO trial also determines the effect of an open lung approach on post-operative extra-pulmonary complications. Finally, in the PROVHILO trial the effect of lung-protective mechanical ventilation is monitored by highly specific biomarkers of lung injury.

## List of abbreviations

AE: Adverse Event; ALI: Acute Lung Injury; AR: Adverse Reaction; ARDS: Acute Respiratory Distress Syndrome; COPD: Chronic Obstructive Pulmonary Disease; EU: European Union; ICU: Intensive Care Unit; I:E: Inspiratory to Expiratory ratio; PBW: Predicted Body Weight; PEEP: Positive end-expiratory pressure; PPC: Post-operative pulmonary complications; RM: Recruitment maneuver; SpO2: Oxyhemoglobin saturation by pulse-oximetry breathing air in supine position; VALI: Ventilator-associated lung injury; WBC: White Blood Cells

## Competing interests

The authors declare that they have no competing interests.

## Authors' contributions

SH: preparation of the initial drafts of the manuscript and preparation of the final version. All: review of the initial drafts of the manuscript. JB: planned the statistical analysis and revised the manuscript. MS, MGdA, PP designed the study, reviewed the initial drafts of the manuscript. All authors approved the final version of the manuscript.

## References

[B1] Putensen C Theuerkauf N Zinserling J Wrigge H Pelosi P Meta-analysis: ventilation strategies and outcomes of the acute respiratory distress syndrome and acute lung injury Ann Intern Med 2009 151 566 576 19841457 10.7326/0003-4819-151-8-200910200-00011

[B2] Tremblay LN Slutsky AS Ventilator-induced lung injury: from the bench to the bedside Intensive Care Med 2006 32 24 33 10.1007/s00134-005-2817-8 16231069

[B3] Papadakos PJ Lachmann B The open lung concept of mechanical ventilation: the role of recruitment and stabilization Crit Care Clin 2007 23 241 250 ix-x. 10.1016/j.ccc.2006.12.001 17368168

[B4] Briel M Meade M Mercat A Brower RG Talmor D Walter SD Slutsky AS Pullenayegum E Zhou Q Cook D Higher vs lower positive end-expiratory pressure in patients with acute lung injury and acute respiratory distress syndrome: systematic review and meta-analysis JAMA 303 865 873 10.1001/jama.2010.218 20197533

[B5] Schultz MJ Haitsma JJ Slutsky AS Gajic O What tidal volumes should be used in patients without acute lung injury? Anesthesiology 2007 106 1226 1231 10.1097/01.anes.0000267607.25011.e8 17525599

[B6] Wrigge H Uhlig U Baumgarten G Menzenbach J Zinserling J Ernst M Dromann D Welz A Uhlig S Putensen C Mechanical ventilation strategies and inflammatory responses to cardiac surgery: a prospective randomized clinical trial Intensive Care Med 2005 31 1379 1387 10.1007/s00134-005-2767-1 16132888

[B7] Wrigge H Uhlig U Zinserling J Behrends-Callsen E Ottersbach G Fischer M Uhlig S Putensen C The effects of different ventilatory settings on pulmonary and systemic inflammatory responses during major surgery Anesth Analg 2004 98 775 781 14980936 10.1213/01.ane.0000100663.11852.bf

[B8] Choi G Wolthuis EK Bresser P Levi M van der Poll T Dzoljic M Vroom MB Schultz MJ Mechanical ventilation with lower tidal volumes and positive end-expiratory pressure prevents alveolar coagulation in patients without lung injury Anesthesiology 2006 105 689 695 10.1097/00000542-200610000-00013 17006066

[B9] Wolthuis EK Choi G Dessing MC Bresser P Lutter R Dzoljic M van der Poll T Vroom MB Hollmann M Schultz MJ Mechanical ventilation with lower tidal volumes and positive end-expiratory pressure prevents pulmonary inflammation in patients without preexisting lung injury Anesthesiology 2008 108 46 54 10.1097/01.anes.0000296068.80921.10 18156881

[B10] Rothen HU Sporre B Engberg G Wegenius G Reber A Hedenstierna G Prevention of atelectasis during general anaesthesia Lancet 1995 345 1387 1391 10.1016/S0140-6736(95)92595-3 7760608

[B11] Jaber S Coisel Y Marret E Malinovsky JM Bouaziz H Ventilatory Management during General Anesthesia: A Multicenter Observational Study Anesthesiology 2006 A1516

[B12] Arozullah AM Daley J Henderson WG Khuri SF Multifactorial risk index for predicting postoperative respiratory failure in men after major noncardiac surgery. The National Veterans Administration Surgical Quality Improvement Program Ann Surg 2000 232 242 253 10.1097/00000658-200008000-00015 10903604 PMC1421137

[B13] Smetana GW Lawrence VA Cornell JE Preoperative pulmonary risk stratification for noncardiothoracic surgery: systematic review for the American College of Physicians Ann Intern Med 2006 144 581 595 16618956 10.7326/0003-4819-144-8-200604180-00009

[B14] Canet J Gallart L Gomar C Paluzie G Valles J Castillo J Sabate S Mazo V Briones Z Sanchis J Prediction of Postoperative Pulmonary Complications in a Population-based Surgical Cohort Anesthesiology 113 1338 1350 10.1097/ALN.0b013e3181fc6e0a 21045639

[B15] Ventilation with lower tidal volumes as compared with traditional tidal volumes for acute lung injury and the acute respiratory distress syndrome. The Acute Respiratory Distress Syndrome Network N Engl J Med 2000 342 1301 1308 10793162 10.1056/NEJM200005043421801

[B16] Crapo RO Morris AH Gardner RM Reference spirometric values using techniques and equipment that meet ATS recommendations Am Rev Respir Dis 1981 123 659 664 7271065 10.1164/arrd.1981.123.6.659

[B17] Crapo RO Morris AH Clayton PD Nixon CR Lung volumes in healthy nonsmoking adults Bull Eur Physiopathol Respir 1982 18 419 425 7074238

[B18] 2005 International Consensus on Cardiopulmonary Resuscitation and Emergency Cardiovascular Care Science with Treatment Recommendations. Part 4: Advanced life support Resuscitation 2005 67 213 247 16324990 10.1016/j.resuscitation.2005.09.018

[B19] Bernard GR Artigas A Brigham KL Carlet J Falke K Hudson L Lamy M LeGall JR Morris A Spragg R Report of the American-European consensus conference on ARDS: definitions, mechanisms, relevant outcomes and clinical trial coordination. The Consensus Committee Intensive Care Med 1994 20 225 232 10.1007/BF01704707 8014293

[B20] Bone RC Toward an epidemiology and natural history of SIRS (systemic inflammatory response syndrome) JAMA 1992 268 3452 3455 10.1001/jama.268.24.3452 1460735

[B21] Thygesen K Alpert JS White HD Jaffe AS Apple FS Galvani M Katus HA Newby LK Ravkilde J Chaitman B Universal definition of myocardial infarction Circulation 2007 116 2634 2653 10.1161/CIRCULATIONAHA.107.187397 17951284

[B22] Bellomo R Ronco C Kellum JA Mehta RL Palevsky P Acute renal failure - definition, outcome measures, animal models, fluid therapy and information technology needs: the Second International Consensus Conference of the Acute Dialysis Quality Initiative (ADQI) Group Crit Care 2004 8 R204 212 10.1186/cc2872 15312219 PMC522841

[B23] Levi M Toh CH Thachil J Watson HG Guidelines for the diagnosis and management of disseminated intravascular coagulation. British Committee for Standards in Haematology Br J Haematol 2009 145 24 33 10.1111/j.1365-2141.2009.07600.x 19222477

[B24] Reintam A Parm P Kitus R Starkopf J Kern H Gastrointestinal failure score in critically ill patients: a prospective observational study Crit Care 2008 12 R90 10.1186/cc6958 18625051 PMC2575570

[B25] Levitt JE Gould MK Ware LB Matthay MA The pathogenetic and prognostic value of biologic markers in acute lung injury J Intensive Care Med 2009 24 151 167 10.1177/0885066609332603 19282296

[B26] Determann RM Wolthuis EK Choi G Bresser P Bernard A Lutter R Schultz MJ Lung epithelial injury markers are not influenced by use of lower tidal volumes during elective surgery in patients without preexisting lung injury Am J Physiol Lung Cell Mol Physiol 2008 294 L344 350 18083770 10.1152/ajplung.00268.2007

[B27] Parsons PE Eisner MD Thompson BT Matthay MA Ancukiewicz M Bernard GR Wheeler AP Lower tidal volume ventilation and plasma cytokine markers of inflammation in patients with acute lung injury Crit Care Med 2005 33 1 6 discussion 230-232. 15644641 10.1097/01.ccm.0000149854.61192.dc

[B28] Del Sorbo L Slutsky AS Ventilatory support for acute respiratory failure: new and ongoing pathophysiological, diagnostic and therapeutic developments Curr Opin Crit Care 16 1 7 10.1097/MCC.0b013e32833500bc 19952735

[B29] Plotz FB Slutsky AS van Vught AJ Heijnen CJ Ventilator-induced lung injury and multiple system organ failure: a critical review of facts and hypotheses Intensive Care Med 2004 30 1865 1872 10.1007/s00134-004-2363-9 15221129

[B30] Arozullah AM Khuri SF Henderson WG Daley J Development and validation of a multifactorial risk index for predicting postoperative pneumonia after major noncardiac surgery Ann Intern Med 2001 135 847 857 11712875 10.7326/0003-4819-135-10-200111200-00005

[B31] Gajic O Dara SI Mendez JL Adesanya AO Festic E Caples SM Rana R St Sauver JL Lymp JF Afessa B Hubmayr RD Ventilator-associated lung injury in patients without acute lung injury at the onset of mechanical ventilation Crit Care Med 2004 32 1817 1824 10.1097/01.CCM.0000133019.52531.30 15343007

[B32] Gajic O Frutos-Vivar F Esteban A Hubmayr RD Anzueto A Ventilator settings as a risk factor for acute respiratory distress syndrome in mechanically ventilated patients Intensive Care Med 2005 31 922 926 10.1007/s00134-005-2625-1 15856172

[B33] Determann RM Royakkers A Wolthuis EK Vlaar AP Choi G Paulus F Hofstra JJ de Graaff MJ Korevaar JC Schultz MJ Ventilation with lower tidal volumes as compared with conventional tidal volumes for patients without acute lung injury: a preventive randomized controlled trial Crit Care 2010 14 R1 10.1186/cc8230 20055989 PMC2875503

[B34] Reis Miranda D Gommers D Struijs A Dekker R Mekel J Feelders R Lachmann B Bogers AJ Ventilation according to the open lung concept attenuates pulmonary inflammatory response in cardiac surgery Eur J Cardiothorac Surg 2005 28 889 895 10.1016/j.ejcts.2005.10.007 16271479

[B35] Pelosi P Rocco PR Airway closure: the silent killer of peripheral airways Crit Care 2007 11 114 10.1186/cc5692 17328793 PMC2151892

[B36] Pelosi P Jaber S Noninvasive respiratory support in the perioperative period Curr Opin Anaesthesiol 23 233 238 10.1097/ACO.0b013e328335daec 20019602

[B37] Lapinsky SE Mehta S Bench-to-bedside review: Recruitment and recruiting maneuvers Crit Care 2005 9 60 65 15693985 10.1186/cc2934 PMC1065091

[B38] Girgis K Hamed H Khater Y Kacmarek RM A decremental PEEP trial identifies the PEEP level that maintains oxygenation after lung recruitment Respir Care 2006 51 1132 1139 17005058

[B39] Talab HF Zabani IA Abdelrahman HS Bukhari WL Mamoun I Ashour MA Sadeq BB El Sayed SI Intraoperative ventilatory strategies for prevention of pulmonary atelectasis in obese patients undergoing laparoscopic bariatric surgery Anesth Analg 2009 109 1511 1516 10.1213/ANE.0b013e3181ba7945 19843790

[B40] Maisch S Reissmann H Fuellekrug B Weismann D Rutkowski T Tusman G Bohm SH Compliance and dead space fraction indicate an optimal level of positive end-expiratory pressure after recruitment in anesthetized patients Anesth Analg 2008 106 175 181 table of contents. 10.1213/01.ane.0000287684.74505.49 18165575

[B41] Reis Miranda D Struijs A Koetsier P van Thiel R Schepp R Hop W Klein J Lachmann B Bogers AJ Gommers D Open lung ventilation improves functional residual capacity after extubation in cardiac surgery Crit Care Med 2005 33 2253 2258 10.1097/01.CCM.0000181674.71237.3B 16215379

[B42] Slutsky AS Tremblay LN Multiple system organ failure. Is mechanical ventilation a contributing factor? Am J Respir Crit Care Med 1998 157 1721 1725 9620897 10.1164/ajrccm.157.6.9709092

[B43] Imai Y Parodo J Kajikawa O de Perrot M Fischer S Edwards V Cutz E Liu M Keshavjee S Martin TR Injurious mechanical ventilation and end-organ epithelial cell apoptosis and organ dysfunction in an experimental model of acute respiratory distress syndrome JAMA 2003 289 2104 2112 10.1001/jama.289.16.2104 12709468

[B44] Dreyfuss D Saumon G Ventilator-induced lung injury: lessons from experimental studies Am J Respir Crit Care Med 1998 157 294 323 9445314 10.1164/ajrccm.157.1.9604014

[B45] Papadakos PJ Cytokines, genes, and ARDS Chest 2002 121 1391 1392 10.1378/chest.121.5.1391 12006416

[B46] Block BM Liu SS Rowlingson AJ Cowan AR Cowan JA Jr Wu CL Efficacy of postoperative epidural analgesia: a meta-analysis JAMA 2003 290 2455 2463 10.1001/jama.290.18.2455 14612482

[B47] Ferreyra G Long Y Ranieri VM Respiratory complications after major surgery Curr Opin Crit Care 2009 15 342 348 10.1097/MCC.0b013e32832e0669 19542885

[B48] Filsoufi F Rahmanian PB Castillo JG Chikwe J Adams DH Predictors and early and late outcomes of respiratory failure in contemporary cardiac surgery Chest 2008 133 713 721 10.1378/chest.07-1028 18263692

[B49] Fernandez-Perez ER Keegan MT Brown DR Hubmayr RD Gajic O Intraoperative tidal volume as a risk factor for respiratory failure after pneumonectomy Anesthesiology 2006 105 14 18 10.1097/00000542-200607000-00007 16809989

[B50] Chawla G Drummond GB Fentanyl decreases end-expiratory lung volume in patients anaesthetized with sevoflurane Br J Anaesth 2008 100 411 414 10.1093/bja/aem376 18216033

[B51] Hedenstierna G Edmark L The effects of anesthesia and muscle paralysis on the respiratory system Intensive Care Med 2005 31 1327 1335 10.1007/s00134-005-2761-7 16132894

